# Spatiotemporal Evolution and Spatial Network Analysis of the Urban Ecological Carrying Capacity in the Yellow River Basin

**DOI:** 10.3390/ijerph19010229

**Published:** 2021-12-26

**Authors:** Ke Liu, Shiwen Yang, Qian Zhou, Yurong Qiao

**Affiliations:** 1 School of Economics and Management, Zhengzhou University of Light Industry, Zhengzhou 450001, China; liuke_liu@163.com (K.L.); hylhyx@163.com (S.Y.); qiaoyurong9@163.com (Y.Q.); 2 Economics School, Zhongnan University of Economics and Law, Wuhan 430073, China

**Keywords:** Yellow River Basin (YRB), ecological carrying capacity (ECC), spatiotemporal evolution, spatial network

## Abstract

Based on the panel data of 82 cities in the Yellow River Basin (YRB) during 2008–2017, this paper calculated the urban ecological carrying capacity (UECC) index by means of the entropy method, drew a spatiotemporal evolution map using ArcGIS10.3 software, used a spatial cold–hot spot model to explore the spatial characteristics of the UECC index, and used the revised gravity model to construct the spatial network of the UECC. In addition, through social network analysis, we obtained the spatial network correlation characteristics of the UECC of 82 cities in the YRB. The study found the following: (1) The UECC index of the cities in the YRB increased steadily, and showed strong non-stationarity in space. The cold and hot spot patterns both changed greatly. Overall, the changes of the hot and cold spots were very significant. (2) The spatial correlation and linkage effects of the UECC in the YRB were not significant. The central cities with higher point centrality and closeness centrality showed the same spatial distribution, and most of them are located in the midstream and downstream of the YRB. The central cities in the midstream and downstream of the YRB had high betweenness centrality, and stood in the center of the association network. (3) The four plates in the spatial correlation network of the UECC in the YRB all showed their advantages and functions. The first plate was the net spillover plate, which was principally allocated in the upstream and midstream of the YRB. The second plate was the broker plate, which was principally located in the midstream and downstream of the YRB, and a few cities in the upper reaches. The third plate was the net inflow plate, which was distributed sporadically in the upstream and downstream of the YRB. The fourth plate was the broker plate, which was scattered in upstream, midstream, and downstream of the YRB. Therefore, it is necessary to shorten the gap of and promote the improvement of the UECC in the YRB.

## 1. Introduction

As the second-longest river in China, the Yellow River stretches across the eastern and central regions; it is a significant ecological safety shelter of China and a key region carrying population activities and economic development. The Yellow River plays an irreplaceable role in national strategic planning and socialist modernization construction. However, with the economic and social development mode of energy, the chemical industry, agriculture and animal husbandry in the YRB in recent years, the phenomena of the excessive exploitation and utilization of water resources, an increasingly fragile ecological environment, and a weak carrying capacity of resources and the environment in the YRB have become increasingly prominent; this has been a constraint on the subject of the development of society and the economy of the YRB. Since the 18th party congress of China, president Xi Jinping, has inspected the ecological protection and economic and social development of the YRB many times. On the morning of 18 September 2019, the President of China hosted a discussion on ecological environment protection and high-quality development in the YRB in Zhengzhou, He-nan Province. He stressed that ecological protection and high-quality development in the YRB were major national strategies. Therefore, we should strengthen the governance of ecological and environmental protection in the YRB, and we should promote high-quality development in the whole watershed.

The YRB is a strategic support belt for China’s economic development in the new era, and its high-quality development is of great significance to the promotion of the high-quality development of China’s economy. In order to promote the ecological protection and high-quality development of the YRB, and to strictly observe the ecological red line, it is necessary to scientifically evaluate the UECC in the basin, and to analyze the relationship and influence of the UECC.

Subsequently, experts and scholars have paid increasingly more attention to the ecological protection of the YRB. In the research on the ecological environment of the YRB, scholars have focused on single-perspective research, such as the effects of water resource management [1], sediment sources [2], and climate change [3] on the natural ecological environment of the YRB. Other scholar shave focused on multi-perspective research, such as the urban agglomerations along the YRB [4], sustainable development [5], coordinated economic and ecological development [6,7], and green industrial development [8]. Overall, research on the ecological conservation and economic growth of the YRB has begun to take shape.

In order to further expand the research on the ecological conservation and economic growth of the YRB, it is necessary to construct a comprehensive evaluation index system to measure the development of the YRB ecosystem. The existing comprehensive measurement of the YRB mainly focuses on several aspects. One aspect is the comprehensive evaluation index system. Some scholars took the three perspectives of the resource-carrying capacity, natural environment and socioeconomic factors as the starting points to analyse the ecological environment quality of the YRB [9]. In order to explore the spatial heterogeneity of the ecological vulnerability of wetlands in the YRB, a study constructed a comprehensive evaluation system and model for the ecological vulnerability of coastal wetlands, and found that the ecological vulnerability of the YRB is generally at a medium level [10]. Other scholars comprehensively evaluated the environmental quality of the middle and upper reaches of the YRB, studied and analysed the environmental status and overall environmental quality of each ecosystem region, and summarized the distribution of the environmental quality in the three regions [11]. Based on a multidimensional perspective, scholars in related fields established a comprehensive evaluation indicator system for the evaluation of urban resilience in the Yellow River basin [12]. The second aspect is the evaluation of the water resource quality in the YRB. Related research analysed the water quality of the three governed rivers of Guijiang, Nenjiang and the lower reaches of the YRB, and established a framework for the measurement of the quality of rivers. The third aspect is the assessment of the soil environmental quality in the YRB [13]. Other scholars evaluated the soil quality of coastal wetlands in the Yellow River Delta using the soil quality index calculated by principal component analysis and the smallest dataset [14]. With further research, some scholars have begun to further deduce the comprehensive measurement of the ecological system level of the YRB in order to study the spatial pattern characteristics. In terms of the spatial and temporal analysis and spatial evolution of the YRB, scholars have mainly studied the changes in the spatial patterns of the land use in the YRB [15], the temporal and spatial matching of water resources and economic development [16], the sedimentation of the Yellow River Delta [17], the spatial pattern analysis of the energy eco-efficiency [18], and the driving mechanism of urbanization development [19]. With the development of the YRB, the flow of production factor resources has become more frequent, and a cross-regional spatial correlation network has gradually formed. Some scholars used the social network analysis method to analyze the spatial network characteristics of urban resilience [20,21]. As a consequence, it is important for the interpretation of the ecological protection and economic development of the YRB from the perspective of spatial networks.

We summarize the above process in Table 1.

Based on the above existing studies, many scholars have explored the YRB from multiple perspectives, such as ecological protection and economic development, the comprehensive measurement of ecosystem levels, spatiotemporal analyses and spatial evolution. Compared with the existing literature, the possible contributions of this article consist in focusing on research on the YRB in the context of the national strategy, constructing a comprehensive evaluation index system for the UECC from a spatial perspective, and systematically analysing the relationship and influence of the UECC in the YRB.

The article establishes the assessment indicator system of the UECC in the YRB. The system consists of a subsystem layer composed of ecological resilience, ecological support capacity and ecological pressure; a criterion layer composed of the resource supply, environmental protection, social progress and economic development; and an index layer composed of the per capita water resources and garden green space. The entropy weight method is used to measure the panel data of 82 cities in eight provinces of the YRB from 2008 to 2017; on this basis, the spatiotemporal evolution is analysed. The social network analysis method analyses the spatial network structure and its linkage effect among cities, and the mutual influences of the UECC are objectively analysed.

## 2. Index System Construction and Research Methods

### 2.1. The Index System Construction

The UECC is usually defined as the ability to provide natural resources for socioeconomic development, and to absorb human waste [22]. First, in order to assess the effectiveness, the UECC is widely used in resource management, city planning and many other social activities [23,24]. Second, compared with single-perspective research, the UECC pays more attention to the integration, sustainability and coordination of the ecosystem from multiple perspectives and wider perspectives. Therefore, it is necessary to use a comprehensive evaluation method to reflect the carrying capacity of the ecosystem of the YRB. Some scholars have established a regional UECC evaluation model that includes the environmental quality, the ecological level, and the social economy. The model includes the coordination capabilities generated by the social and economic subsystems in the ecosystem, and the resources and environment subsystems contribute to the healthy operation of the system. Using the support capacity and the ecological pressure generated by the economic growth subsystem to analyse the change characteristics of these elements is an effective means to evaluate the sustainable development status of a region [25]. Therefore, a comprehensive evaluation index system for the UECC of the YRB is constructed from multiple perspectives, including ecological resilience, ecological support, and ecological pressure. In the process of constructing the index system, it is also necessary to consider the availability of data.

Regarding ecological resilience, some scholars believe that the expansion of space will first affect land and biological resources [26]. Moreover, improper urban development will affect farmland and wetlands, etc., and then affect the ecological environment [27]. Some scholars also believe that the scale and intensity of urban development should not exceed the certain carrying capacity of a specific regional ecosystem [28]. Therefore, the unlimited expansion of the population will break the ecological carrying limit, and the balanced relationship between the population and land should also be included in the evaluation index. Therefore, the proportion of urban construction land, land productivity, and land area per capita were selected as evaluation indicators to measure ecological resilience.

Regarding ecological support, some scholars have introduced life cycle assessment methods to estimate the level of ecological deficits in a region. The assessment factors include the environmental impacts of the products produced by human activities, including raw materials and waste [29]. Therefore, automatic maintenance and regulation in the ecosystem, the expansion of resources and the environment, and the maintenance of the intensity of social and economic activities have become important measurement factors for the evaluation of the UECC. Therefore, the comprehensive utilization rate of industrial solid waste and the harmless treatment rate of domestic waste were selected as the evaluation indicators of the environmental protection criterion. In addition, the socioeconomic level of a region and the intensity of environmental governance also have great impacts on the UECC. It was concluded that the degree of urbanization and economic development can have important impacts on the local ecosystem and energy use. Accelerating the urbanization process and diversifying economic sources are effective ways to increase the energy footprint [30]. The energy structure, energy intensity and land structure of a region have an impact on the ecological pressure of the region [31]. In addition, social economy, education, secondary industry, and the living environment are the key influencing factors of urbanization which have an impact on the urban environment [32]. In spite of the points of view of research and the studying methods being different, the majority of scholars believe that the impacts of scientific and technological progress social systems and lifestyles in human production on the UECC cannot be ignored. Therefore, we chose standard indicators commonly used in the literature, such as the number of doctors per 10,000 people, and urban maintenance and construction fund expenditures. These can measure the social progress and policy support level of a city.

Regarding ecological pressure, some scholars have proposed that there is a close relationship between social systems and ecological functions within a region. Therefore, it is feasible to evaluate the pressure on the ecological environment of a region by considering the pressure of human activities on the ecosystem in the region [33]. China is still in a period of rapid economic growth. Judging from the environmental Kuznets inverted “U”-shaped curve, China is still on the left side of the inflection point of the inverted “U” curve of the environmental Kuznets curve. Various economic growth activities will further increase the environmental pollution. Therefore, many pressure factors—such as increasing environmental pollution, huge resource demand and congestion diseconomies in general, etc.—appear and occupy dominant positions. These pressure factors will damage the urban ecological environment and bring great pressure to the sustainable development of a region [34,35,36,37]. In addition, China’s household registration (hukou) system is the main means to control population mobility. The household registration system can reduce rural-to-urban migration, ease the pressure on the urban population, and have a beneficial impact on the improvement of the UECC in the YRB [38]. Therefore, environmental damage and population pressure have become the main limiting factors in the measurement of the comprehensive UECC. We use indicators such as industrial sulfur dioxide emissions to measure the environmental damage of the UECC, and indicators such as population density to measure population pressure. The specific variables are shown in Table 2.

### 2.2. Data Processing

#### 2.2.1. Data Source and Processing

Based on the data consistency, availability and operability, the samples of this paper are from 82 cities in the YRB from 2008 to 2017. There are 37 indicators for each city. The research data mainly came from the China City Statistical Yearbooks. In order to ensure data continuity and facilitate the analysis, we chose the interpolation method to fill the individual missing data and eliminate Haidong city due to it possessing too much missing data.

#### 2.2.2. Entropy Method

The entropy method provides a basis for multi-dimensional comprehensive evaluation, which is more objective and avoids the influence of human factors in subjective evaluation [39]. Here, the entropy method is used to calculate the weights of different evaluation indexes in the evaluation index system of the UECC in the YRB. The average values of these weights from 2008 to 2017 are listed in the sixth column of Table 2. The urban comprehensive index coefficients are calculated by using the weights.

For the elimination of the effect of different dimensions of indicators, we adopted data standardization using the range method, and the indicators were divided into positive indicators and negative indicators. The calculation equation are as follows:

Positive indicators:

(1) $$x_{ij}^{\prime }=\frac{x_{ij}-\min(x_{ij})}{\max(x_{ij})-\min(x_{ij})}$$

Negative indicators:

(2) $$x_{ij}^{\prime }=\frac{\max(x_{ij})-x_{ij}}{\max(x_{ij})-\min(x_{ij})}$$

In Equations (1) and (2), the original value of the *i*-th index in year *j* is expressed as $x_{ij}$, the maximum value of an index is expressed as $\;\max(x_{ij})$, the minimum value of an index is expressed as $\min(x_{ij})$, and $x_{ij}^{\prime }$ is the index obtained after the standardization result.

Calculate the proportion of the *j*-th index value in year *i* as

(3) $$Y_{ij}=\frac{X_{ij}}{\sum_{i=i}^{m}X_{ij}}$$

Compute the index information as

(4) $$e_{j}=-k\overset{m}{\underset{i=1}{\sum }}\left(Y_{ij}\times lnY_{ij}\right)$$

Compute the entropy of information redundancy as

(5) dj=1−ej

Compute the indicator weight as

(6) $$W_{i}=\frac{d_{j}}{\sum_{j=i}^{n}d_{j}}$$

Compute the evaluation score of the indicators as

(7) $$S_{ij}=W_{i}\times X_{ij}$$

In Equations (3)–(7), $x_{ij}$ represents the value of the *j*-th evaluation index in the *i*-th year; $\min(x_{ij})$ and $\max(x_{ij})$ are the minimum and maximum values of the *j*-th evaluation index in all years, respectively, $k=\frac{1}{\ln m}$, *m* is the number of evaluation years and *n* is the number of indices.

### 2.3. Research Method

#### 2.3.1. Spatial Exploration Method of the Spatial ECC Index

Exploratory spatial data analysis (ESDA) is a method to reflect the special dependence or spatial heterogeneity of geographical phenomena. It generally takes spatial data distribution characteristics as the breakthrough points and reflects the regional adjacency relationship through the spatial weight matrix [40]. It is generally used to study spatial data distribution characteristics.

First, in order to research the spacial correlation and difference of the UECC, and to analyse the spatial agglomeration trend of 82 cities in the YRB, this paper selected the global Moran’s I index. The equation is as follows:

(8) $$I=\frac{\sum_{i=1}^{n}\sum_{j=1}^{n}w_{ij}\left(x_{i}-\bar{x}\right)\left(x_{j}-\bar{x}\right)}{S^{2}\sum_{i=1}^{n}\sum_{j=1}^{n}w_{ij}}$$

This paper selected the Getis-Ord Gi* index to describe the special distribution features of hot spots and cold spots, and explored the local spatial correlation and degree of difference of the ECC. The specific calculation equation is as follows:

(9) $$G_{i}^{*}=\frac{\sum_{j=1}^{n}w_{ij}\left(d\right)x_{j}}{\sum_{j=1}^{n}x_{j}}$$

In Equations (8) and (9), *I* is the global Moran’s I; *n* is the number of cities;$\;x_{i}$ and $x_{j}$ represent the UECC of city *i* and city *j* separately; and $w_{ij}$ is the special weight matrix. The Z test is conducted on Gi*. If the value of Z(Gi*) is positive, the value around position *i* is greater than the average value, which indicates a high-value spatial concentration (hot spot area); otherwise, it indicates a low-value spatial concentration (cold spot).

#### 2.3.2. Spatial Correlation Network Method

In the construction of a spatial association network, the establishment of a spatial association network is the premise and foundation of social network analysis. Therefore, we seek to set up a spatial association network of the UECC in the YRB. According to spatial correlation network theory, in this paper, the spatial association network of the UECC in the YRB is based on the collection of regional relations. In the network, cities are the “points”, and the spatial correlation between cities is the “line”. The composition of these points and lines is the spatial correlation network of the UECC in the YRB. Simultaneously, in order to prevent the problem of the VAR model being too sensitive to the selection of the lag order, which leads to the imprecise characteristics of the network structure, this paper considers the factors of the economic development and geographical distance of each city. The gravity model is introduced into the spatial correlation network in order to objectively reveal the evolutionary characteristics of the spatial correlation. Furthermore, with the purpose of enhancing adaptability, the gravity model is corrected. The corrected gravity model refers to related literature [21]. With the correction, the gravity model is:

(10) $$F_{ij}=r_{ij}\frac{\sqrt{R_{i}\times GDP_{i}}\sqrt{R_{j}\times GDP_{j}}}{{\left(\frac{D_{ij}}{agdp_{i}-agdp_{j}}\right)}^{2}},r_{ij}=\frac{R_{i}}{R_{i}+R_{j}}\;0\leq i,j\leq 82$$

The type represents the UECC relationship between city *i* and city *j*. *R_i_*, *GDP_i_*, and *agdp_i_* denote the UECC, GDP, and per capita GDP of city *i*. *D_ij_* means the distance between city *i* and city *j*, and denotes the rate of contribution of city *i* to the relationship of the UECC between city *i* and city *j*.

In the feature analysis of the spatial correlation global network of the UECC in the YRB, the analysis of the spatial correlation network characteristics usually requires the overall network density, degree of network correlation, network diameter, and average path length. In the spatial correlation network analysis, on the basis of the data of the global network density, the degree of closeness of the spatial correlation network of the UECC in the YRB can be obtained. The larger the overall network density is, the closer the relationship between the cities, and the greater the impact on the UECC network structure in different cities. The degree of network association shows the vulnerability and robustness of the spatial correlation network of the UECC in the YRB. If there is a direct correlation path between one of the cities and many other cities, the overall degree of correlation of the network will increase. However, at the same time, the spatial network is highly dependent on the city, and the stability of the overall spatial network is low. The degree of correlation in the spatial correlation matrix is usually represented by the network diameter and average path length.

For the analysis method of network individual centrality characteristics, in social network analysis, the central characteristics of individual networks are usually reflected in the main network indicators, such as the point centrality, closeness centrality, and betweenness centrality. Point centrality reflects the number of cities directly associated with city *i* in the network. The greater the degree of point centrality is, the greater the number of cities that are associated with city *i,* and the higher the central position of city *i* in the network. Betweenness centrality reflects the degree to which city *i* is an “intermediary” in the spatial network, which is the extent to which the city plays the role of an “intermediary” and a “bridge”. The greater the degree of betweenness centrality is, the closer the location of city *i* is to the network center, and the greater the bridge’s role in communicating with each city. The closeness centrality is used to reflect the distance between them. The higher the closeness centrality, the closer city *i* is to other cities.

Regarding the module analysis method of the spatial correlation network, module analysis can describe the internal structure for different spatial network roles, and can determine the relationship and linkage between different plates. Therefore, this paper divides the network space of the UECC in the YRB into three types: the net inflow plate, the net spillover plate, and the broker plate. The first type is the net inflow plate. The plate members come from the relationships between the members within the plate; this plate also accepts the relationships between members from other plates, and the number of relationships sent by this plate is far less than that received from other plate members. The second type is the net spillover plate. The number of relations of the net spillover plate to other plates is significantly greater than that accepted by this plate from other plates. The last type is the broker plate. The plate members accept the relationships from other plate members and the relationships from internal members. However, the number of relationships coming from within the plate is less, and the plate mainly plays the role of an intermediary and a bridge.

## 3. Analysis of the Spatiotemporal Evolution of the UECC in the YRB

**Figure 1:**
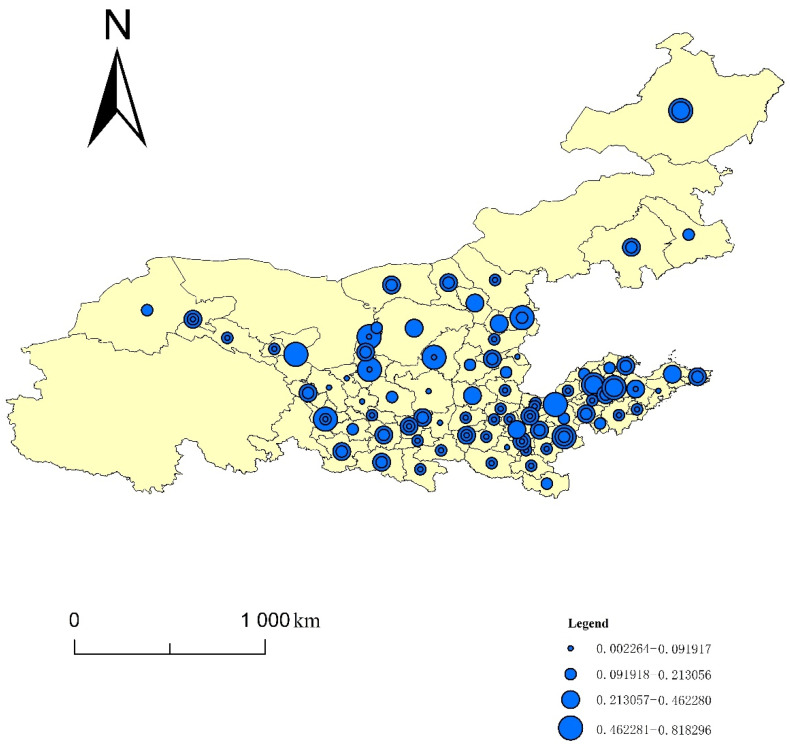
Spatiotemporal evolution of the urban ecological resilience in the YRB.

**Figure 2:**
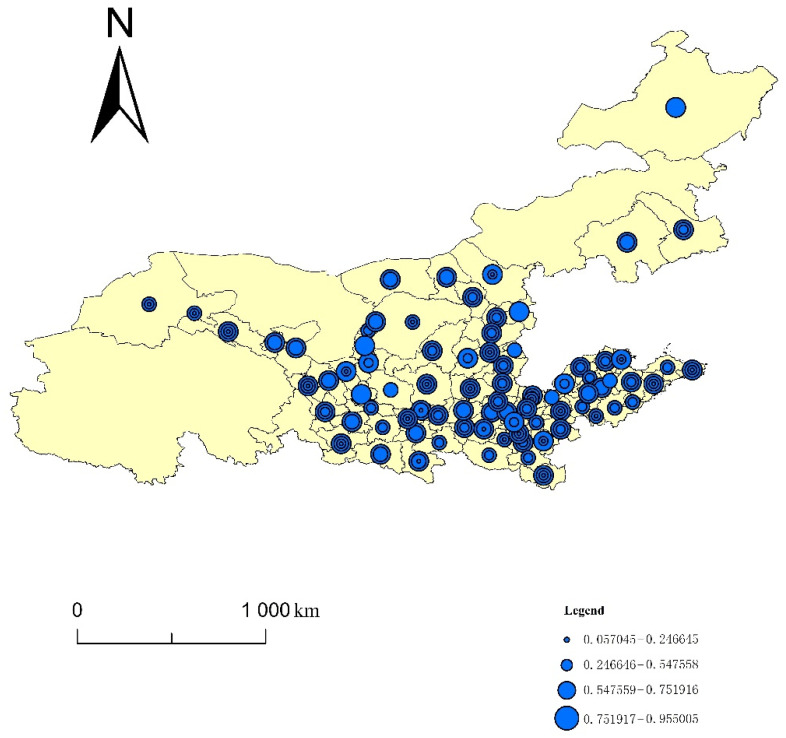
Spatiotemporal evolution of the urban ecological pressure in the YRB.

**Figure 3:**
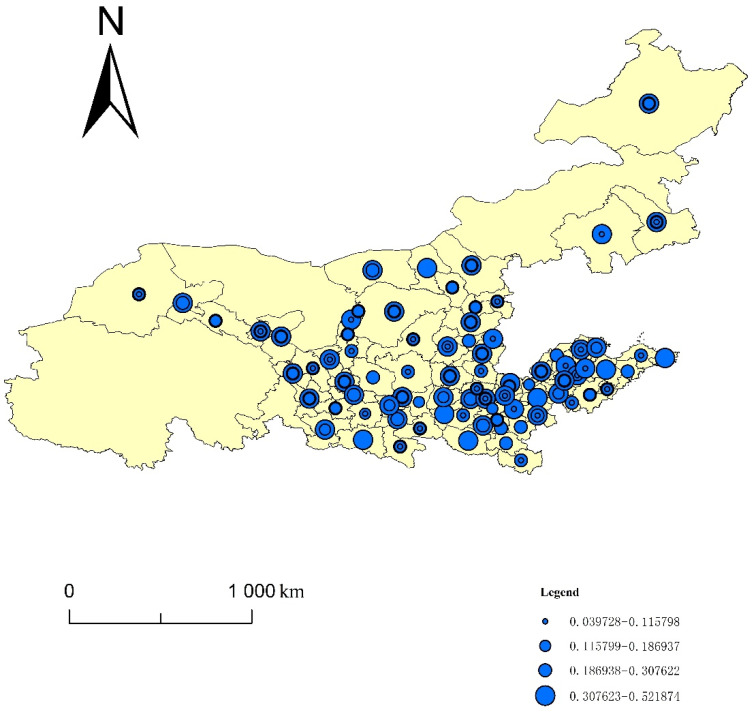
Spatiotemporal evolution of the urban ecological support capacity in the YRB.

**Figure 4:**
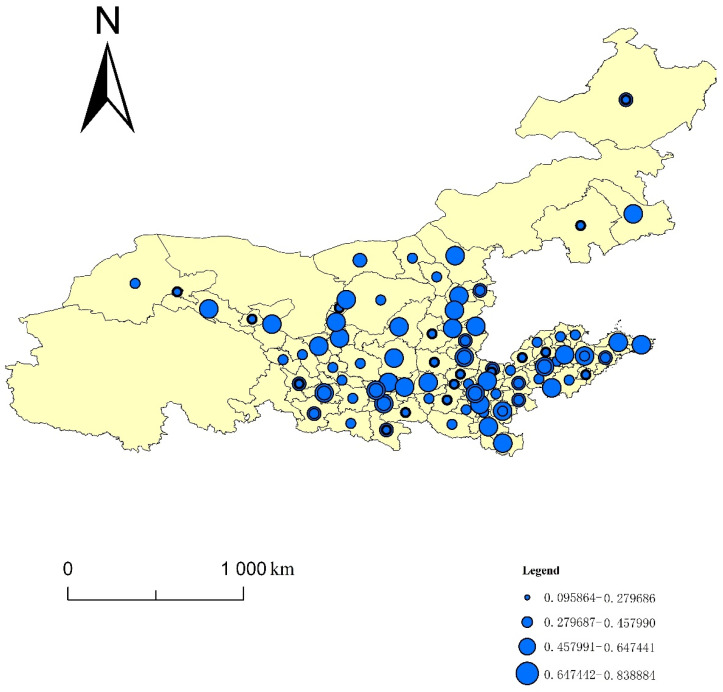
Spatiotemporal evolution of the UECC in the YRB.

Using the entropy method, this paper calculated the four indices of the ecological resilience, ecological pressure, ecological support and UECC of 82 cities in eight provinces of the YRB from 2008 to 2017. Using ArcGIS10.3, this paper generated a spatiotemporal evolution map using the natural segment grading method (Figure 1, Figure 2, Figure 3 and Figure 4). The natural segment grading method maximizes the similarity of the internal indexes of each level and the maximum dissimilarity between each level, and takes into account the range and number of each level of index as much as possible. In the figures, the size of the circle where each city is located denotes the variation in the distribution range of the index, which can give the strength of the non-stationarity in space. The size and number of overlapping circles within a circle represent the situation and number of indices covering the distribution interval of a city during the study period, and reflect the strength of the non-stationarity over time.

### 3.1. Spatiotemporal Evolution Analysis of the Urban Ecological Resilience in the YRB

In order to compare the distribution of the high and low values of the ecological resilience index of the cities in the YRB, as well as the changes of the index over time, the following analysis was made according to Figure 1: the index is between 0.002264 and 0.818296, showing a strong nonstationary spatial distribution. The map shows that the distribution of high-value cities is comparatively concentrated downstream. Furthermore, low-value cities, with occasional high-value cities, are comparatively concentrated in the upstream and middle reaches. Specifically, cities in the middle and upper reaches, such as Wuwei, Yulin and Yuncheng, and cities in the lower reaches, such as Jinan, Yantai, Weifang and Weihai, have relatively strong ecological resilience. Besides this, Yinchuan (upper reaches), Ordos, Linfen, Hohhot, Chifeng and Hanzhong (middle reaches), and Zhengzhou (lower reaches) also show good ecological resilience. There are two possible reasons for this distribution of the total value of the urban ecological resilience in the YRB in Figure 1. First, regarding the natural environment, the plains of downstream cities of the YRB are broad, the climate is suitable, and the four seasons are distinct. There are many wetland ecosystems in the lower reaches of the YRB. The ecological environment construction has good natural conditions. Second, regarding humanistic conditions, cities in the lower reaches of the YRB have developed traditional agriculture, numerous industries, strong comprehensive economic strength, and an economic foundation to support the construction of ecological civilization. In particular, the downstream cities in Shandong and Henan have accumulated rich experience in the long-term flood control and flood resistance process. In recent years, many measures have been taken to utilize water resources, land and spatial development, and ecological protection and restoration; remarkable results have been achieved.

From the perspective of changes in the ecological resilience index of cities in the YRB over time, the temporal dimension distribution shows that the ecological resilience index of 54 cities spans several intervals and presents a scattered distribution trend in space. The specific cities include Jiayuguan, Dingxi, and Yinchuan upstream; Datong, Chifeng, and Xianyang midstream; and Shangqiu, Xinxiang, and Jinan downstream. Most of them show an increasing trend of ecological resilience, indicating continuous improvement and optimization.

### 3.2. Spatiotemporal Evolution of the Urban Ecological Pressure in the YRB

In order to compare the distribution of the high and low values of the ecological pressure index of the cities in the YRB and the changes of the index over time, the following analysis was made according to Figure 2. The ecological pressure index is distributed between 0.057045 and 0.955005, showing a certain degree of spatial distribution stability. The ecological pressure index of most cities in the YRB is high, among which Guyuan, Qingyang, Jiayuguan and Dingxi in the upper reaches, and Datong in the middle reaches have the highest ecological pressure. A small number of cities, such as Ankang, Chifeng, Qingdao, and Rizhao downstream, have low ecological pressure indices. The main reason for this distribution is that most cities in the upstream and midstream of the YRB are undeveloped, and possess population poverty and unreasonable industrial structures. Some areas—such as the Taihang Mountain area, Lvliang Mountain area, Lvpan Mountain area, and Southeast Qinghai–Northwest Sichuan Tibeta—are widely distributed, with insufficient infrastructure construction, imperfect livelihood development, and low incomes for a long time. Moreover, cities in the upstream and midstream of the YRB have long been dominated by extensive and inefficient traditional industries. Emerging industries are missing. Industrial development occurs at the expense of resources and environmental protection. The problems of low quality and low efficiency are prominent. Excellent talent and a large number of funds flow out. It is difficult to balance ecological protection and economic development, and high-quality development is insufficient.

From the perspective of changes in the ecological pressure index of cities in the YRB over time, the ecological pressure index of 52% of the cities spans several intervals, and there is a certain time nonstationary effect. These cities include the upstream cities of Lanzhou, Baiyin, Zhongwei, and Longnan; the midstream city of Ulanqab; and the downstream cities of Zhengzhou, Heze, Weifang, and Jining. This phenomenon may be due to the acceleration of urbanization in recent years, the rising urban population, and the pressure on the local ecological environment having had different impacts.

### 3.3. Spatiotemporal Evolution Analysis of the Urban Ecological Support Capacity in the YRB

In order to compare the distribution of the high and low values of the ecological support capacity index of the cities in the YRB, and the changes of the index over time, the following analysis was made according to Figure 3. The value is between 0.039728 and 0.521874, showing a strong nonstationary spatial distribution. The distribution of high-value cities downstream of the YRB is comparatively concentrated, and the distribution of high-value cities in the middle and upper reaches of the YRB is relatively decentralized. Specifically, Baotou and Hulunbuir in the middle reaches, and Jinan, Taian, Binzhou, Dongying, Heze, and Xinxiang downstream have higher ecological support capacity indices. Regarding the spatial pattern of economic and social development, due to the accelerated construction of urban agglomerations and national key development zones in the YRB in recent years, the ecological support capacity of the Shandong Peninsula urban agglomeration centered on Jinan and Qingdao; the Zhongyuan urban agglomeration centered on Zhengzhou, Luoyang and Kaifeng; and the national key development zones centered on Guanzhong-Tianshui and Hohhot-Baotou-Ordos-Yulin has increased. The corresponding cities in these areas have prominent geographical advantages, moderate geographical locations, rich resources and well-developed urbanization, which play irreplaceable roles in the stimulation of economic and social development, and the improvement of the UECC of the YRB.

From the perspective of the changes in the ecological support capacity index of cities in the YRB over time, the ecological support index of 66 cities changed greatly from 2008 to 2017. Among them, nearly 80% showed an increase in ecological support. The ecological support indices of some cities (such as Jinchang, Lvliang, Tongliao, Binzhou, Taian, Shangqiu, and Xinxiang) crossed more than three intervals to achieve leapfrog improvements. This phenomenon reflects the fact that, in recent years, with the implementation of a series of environmental protection measures—such as national returning farmland to forests, returning grazing lands to grasslands, and the 3-North Shelter Forest Program, as well as the further consolidation of the status of major grain-producing areas with the North China Plain, Fenwei Plain and Hetao Plain as the carriers and the energy-rich areas dominated by Shanxi and the Ordos Basin—the ecological environment has been continuously improved.

### 3.4. Spatial Analysis of the UECC in the YRB

In order to compare the distribution of the high and low values of the UECC index of the cities in the YRB, and the changes of the index over time, the following analysis was made according to Figure 4. The UECC index of the YRB is between 0.095864 and 0.838884, showing a certain degree of a nonstationary spatial distribution. The UECC index of most cities in the midstream and downstream of the YRB—including Weihai, Yantai, Weifang, Taian, Zaozhuang, Xinxiang and Zhengzhou—is high. The UECC of some cities in the upper reaches of the YRB—including Jiuquan, Jiayuguan, Jinchang, Baiyin, Qingyang and Pingliang—is low. The results show that the UECCs of cities in the midstream and downstream of the YRB are higher than those of the upstream cities. This phenomenon is due to the lack of water resources upstream of the YRB. Upstream cities are mostly typical alpine ecosystems, with perennial drought and less rain. The Sanjiangyuan Mountains, Qilian Mountains, plateau and glaciers are vulnerable to the ecological environment in China. Coupled with the low degree of economic development of most cities, the upstream cities in the YRB are not dominant in ecological protection and economic development. However, the ecological conditions in the lower reaches of the YRB were good. Additionally, due to the cities in the lower reaches of the YRB relying on location and policy advantages in the early stage, their economic foundation is relatively advanced, their infrastructure construction is relatively perfect, and their people’s awareness of environmental protection is strong. The overall UECC of the local cities occupies a leading position.

From the perspective of changes in the UECC index of cities in the YRB over time, there are approximately 34 cities with a UECC index across multiple intervals, and the majority of the cities show an increase in their UECC. Specifically, these cities include upstream cities such as Jiayuguan, Jinchang, Dingxi and Longnan; midstream cities such as Jinzhong; and downstream cities such as Ankang, Datong, Zhoukou, Qingdao and Heze. This indicates that China has achieved remarkable results in various ecological restoration and protection measures in the YRB. Important agricultural and animal husbandry production and energy bases in China have been further consolidated. People‘s living has undergone the significant improvements, and the UECC in the YRB is steadily improving.

## 4. Cold and Hot Spot Analysis of the UECC in the YRB

**Figure 5:**
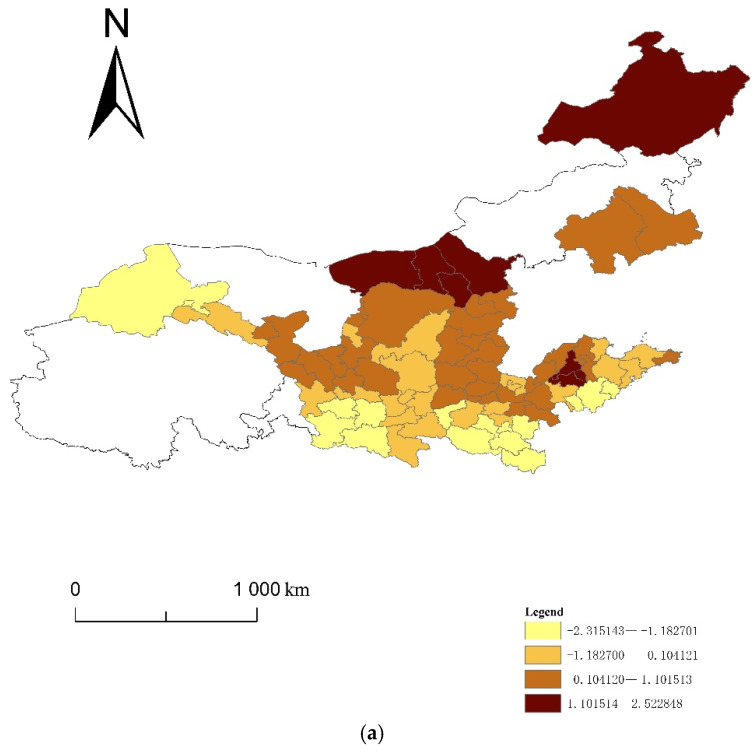

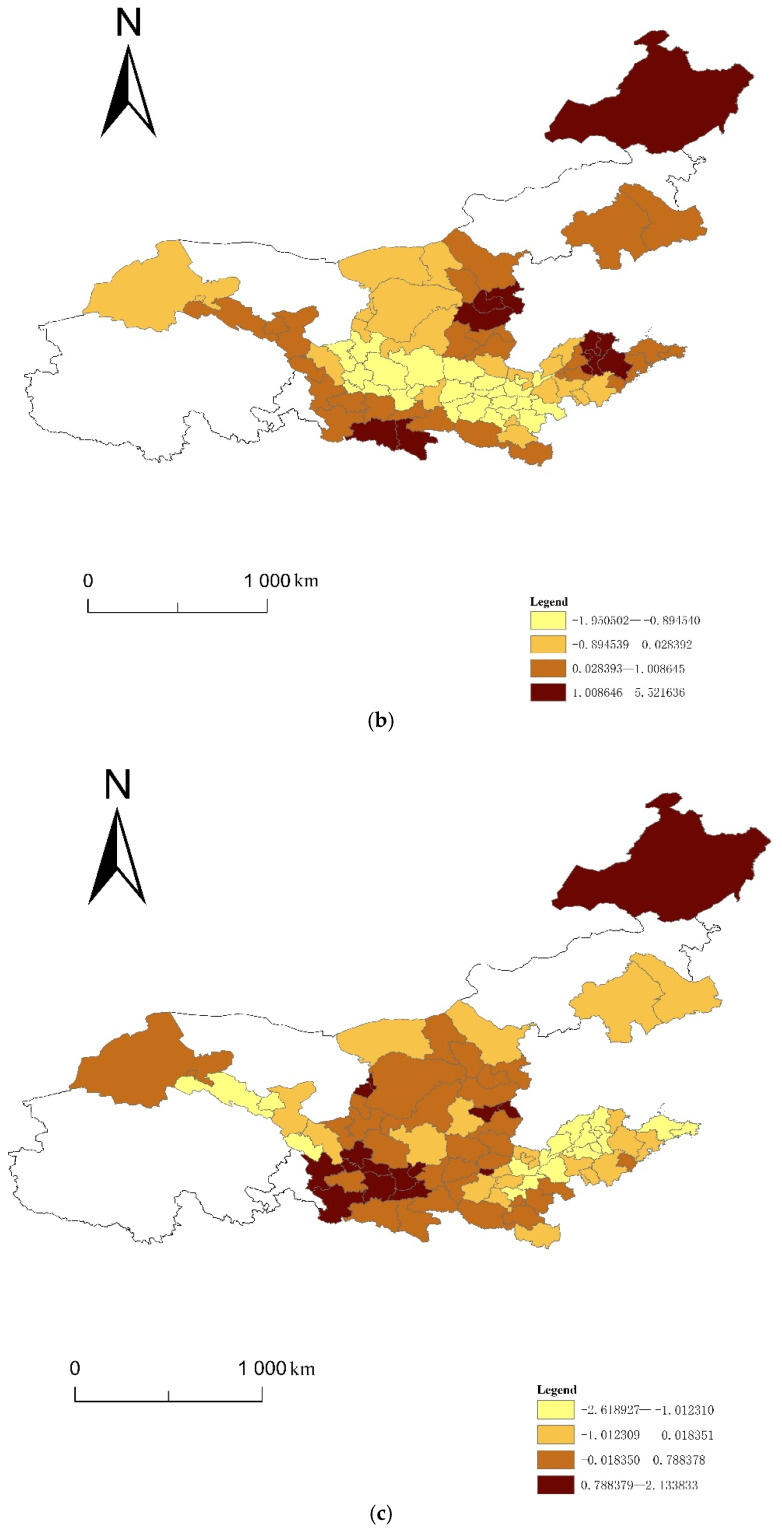
(**a**) Cold–hot evolution diagram of the UECC in the YRB in 2008. (**b**) Cold–hot evolution diagram of the UECC in the YRB in 2012. (**c**) Cold–hot evolution diagram of the UECC in the YRB in 2017.

In order to quantitatively analyse the spatial and temporal evolution characteristics of the UECC in the YRB, the global Moran’s I index of the UECC in the YRB from 2008 to 2017 was calculated using the spatial autocorrelation model. The values were 0.0657, −0.0819, 0.0207, 0.1327, 0.0759, 0.0164, −0.0215, −0.0585 and −0.0347, respectively, which are significant at the 1% significance level. The results showed that the UECC in the YRB was negatively correlated with spatial autocorrelation, and there was a geographical dispersion effect. Although the global spatial autocorrelation model can judge the whole spatial correlation, it cannot differentiate the spatial autocorrelation in different cities. Therefore, in order to further study the evolutionary characteristics of the local spatial agglomeration of the UECC in the YRB, and to consider the differences in the sample time length and information changes in different years, we used ArcGIS10.3 to calculate the local Gi* indices of the cities in 2008, 2012 and 2017. According to the results, the natural fracture method was adopted to divide the spots into hot spots, cold spots, sub-hot spots and sub-cold spots. We drew cold–hot evolution diagrams of the UECC in the YRB, as shown in Figure 5a–c.

Overall, the pattern of cold and hot spots of the UECC in the YRB changed greatly from 2008 to 2017, and the change in cities with different cold and hot spots was considerable. Specifically, regarding the evolution of hotspots and sub-hot spots, most of these spots were located in the midstream and downstream of the YRB in 2008, and there was a sporadic distribution of cities in the upper reaches of the YRB. Specifically, these cities include downstream cities such as Jinan, Taian, Laiwu, Heze, Kaifeng and Shangqiu, and cities in the middle reaches such as Bayannaoer, Baotou, Ulanqab, Hohhot, Hulunbeir, Ordos, Chifeng and Tongliao. This phenomenon was due to the Shandong Peninsula in the lower reaches of the YRB relying on location, policy advantages, and transportation hubs. Due to the area’s leading economic foundation, its infrastructure construction is relatively perfect, and the overall UECC occupies a leading position, which plays a leading role in the surrounding cities. In 2012, the number of hot spots and sub-hot spots of cities in the upper and middle reaches gradually increased to 37 cities, including upstream cities such as Zhangye, Jinchang, Wuwei, Lanzhou, Dingxi, and Longnan; midstream cities such as Hulunbeir, Hanzhong, Ankang, Ulanqab, Datong, and Shuozhou; and downstream cities such as Binzhou, Dongying, and Weifang. The hot spots and sub-hot spots expanded further in 2017, including upstream cities such as Dingxi, Guyuan, Longnan and Pingliang; midstream cities such as Baoji, Taiyuan and Yangquan; and downstream cities such as Jiaozuo, Nanyang and Zhumadian. This phenomenon shows that urban agglomerations—such as the Zhongyuan Urban Agglomeration, the Guanzhong Plain Urban Agglomeration, the Lanxi Urban Agglomeration, the Jinzhong Urban Agglomeration, and the Hohhot-Baotou-Ordos-Yulin Urban Agglomeration—have begun to take shape. The economic development and environmental protection of the central cities have led to the development of the surrounding cities, and the level of the UECC has increased.

Regarding the evolution of the cold and sub-cold points, in 2008, these points included 38 cities primarily allocated in the upstream and midstream of the YRB, including Jiuquan, Jiayuguan, Zhangye, Longnan, Tianshui, Hanzhong, Nanyang, Xinyang, Zhumadian, and Linyi. By 2012, the cold and sub-cold points began to transfer to the downstream of the YRB. The upstream YRB cold points changed to sub-cold points. Compared with 2008, the range of cold points and sub-cold points in the YRB slightly expanded to 45 cities, including upstream cities such as Wuzhong and Qingyang; middle reach cities such as Yanan, Weinan and Yuncheng; and downstream cities such as Luoyang, Zhengzhou, Zhoukou, Linyi and Heze. This phenomenon is due to the impact of the global financial crisis in 2008. The level of social and economic development will have a certain impact on the UECC [18]. Therefore, from 2008 to 2012, the economic development in the middle reaches of the YRB was poor, and ecological protection was weak. Therefore, the ability to improve the UECC of the surrounding cities was weakened. By 2017, the range of cold spots and sub-cold spots in the YRB narrowed again. Several cities in the midstream and upstream of the YRB changed from sub-cold spots to sub-hot spots, and from cold spots to sub-cold spots, or even sub-hot spots. Only 17 cities were cold spots, and most other cities changed to sub-cold spots, sub-hot spots and hot spots. It can be concluded that the development level of the UECC in the YRB in 2017 showed a balanced trend.

## 5. Spatial Correlation Network Characteristics and Dynamic Evolution of the UECC in the YRB

**Figure 6:**
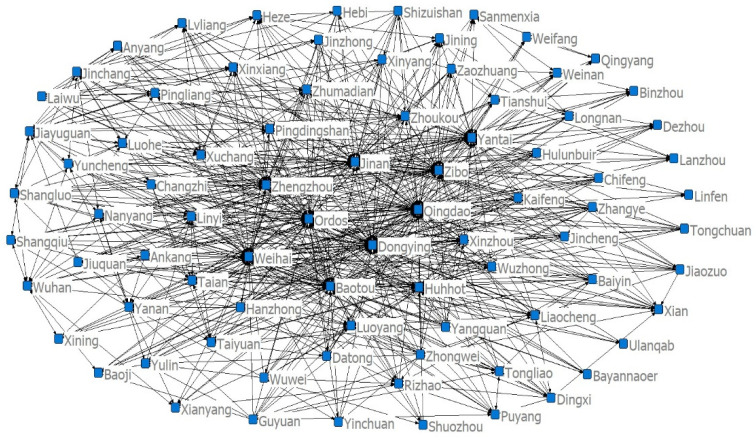
Spatial correlation network diagram of the UECC in the YRB.

First, according to Equation (10), the gravity matrix of the UECC in the YRB is calculated, and then it is transformed into an asymmetric matrix of the spatial correlation network. The specific conversion standard is as follows: the mean of each row in the gravity array is calculated, and then other values are compared with the average value. If the gravity value is greater than the average value of the row, it is denoted by 1, indicating that the row city is correlated with the column city. If the gravity value is lower than the average value, it is denoted by 0, indicating no correlation between the row cities and column cities. Simultaneously, we took 2017 as an example, imported the asymmetric matrix of the spatial correlation network into the UCINET software and the spatial correlation network diagram of the UECC in the YRB, which shows the structure plotted by using the visualization tool NetDraw (Analytic Technologies, Lexington, KY, USA) (Figure 6). This figure presents a “scale-free feature”, and the heterogeneity is obvious. A few cities have more spatial connections than other cities, and most cities have fewer spatial connections. Therefore, the correlation among the cities is generally low. Only when external shocks have a great impact on a few cities with high correlation can they impact all of the cities in the YRB, indicating that the spatial network of the UECC in the YRB has certain vulnerability.

In order to further explore the spatial correlation network features of the UECC in the YRB, the average path length, number of relationships, average number of relationships, network density and degree of network correlation were analysed (Table 3). First, the maximum value of the average network length was 2.258, which is much lower than the total number of cities in the network (82), and there was an obvious “small world phenomenon”. Second, the degree of network correlation fluctuated from 0.8794 to 0.9637 during the study period. This indicates that most of the cities in the YRB are connected, while some cities are independent of the network. The connectivity between the network nodes in general and the overall spatial correlation and linkage effect is not obvious. Finally, regarding the network density and number of relationships, the process of the network density had a slight change, and the maximum value is only 0.1659, indicating that the spatial correlation was still at a low level. The average actual number of relationships was 1022, and there was still a large gap with the maximum value of 6642 (81*82), meaning that there is considerable room for improvement.

### 5.1. Individual Network Characteristics of Spatial Association

In order to study each member’s status and role in the network, we mainly studied individual networks’ centrality. Centrality characteristics need to be measured by three indicators: point centrality, closeness centrality and betweenness centrality. However, after calculating the centrality index of the network, it was found that the index of each city had no significant change, and the relative positions did not change significantly. Considering the accumulation of ecological environmental governance and economic construction achievements in cities, this paper only analysed the results from 2017.

#### 5.1.1. Point Centrality

The measurement and calculation show that the average point centrality was 21.319, and 16 parameter values were higher than the mean value, indicating that the cities represented by these parameters are more related to other cities, which are relatively concentrated. Furthermore, the stability of the overall network structure is also highly dependent on these cities. Among these cities, cities with point centrality exceeding 80% included upstream cities such as Jiayuguan; midstream cities such as Hohhot, Ordos, and Baotou; and downstream cities such as Dongying, Weihai, Qingdao, and Zibo. There were a total of 16 cities. These cities had linkage effects with other cities, indicating that they are at the center of the linkage of the UECC in the YRB. The reason may be that they are mostly provincial capital centers or important energy-rich areas with abundant resources, obvious location advantages, and high levels of urbanization. They are the main carriers of the population and productivity distribution in the YRB. Moreover, in upstream cities such as Qingyang, Xining, and Yinchuan; midstream cities such as Bayannaoer, Ulanchabu, and Shuozhou; and the downstream city of Weifang, the degrees of the centralities in the associated network are relatively low. They are less related to other cities. The reason may be that these cities are restricted by geographical and economic conditions, and their ECCs are not closely related to other cities in space.

The spatial network of the UECC in the YRB was further investigated using the point-out-degrees and point in-degrees. The mean value of the out-degree and in-degree is 11.329, and the out-degrees of 41 cities were greater than the mean value. The top cities were the upstream cities of Jinchang and Jiayuguan, the midstream cities of Baotou and Ordos, and the downstream cities of Zhengzhou and Xuchang, indicating that the spillover effects of these cities are obvious. The remaining cities will be affected by these cities. Additionally, there are 18 cities with a point in-degree greater than the average, and the corresponding point centrality rankings of these cities are also relatively high. These cities are mostly located in areas with good industrial foundations and developed economies. Due to the siphoning effect in the development and construction of these cities, there are different degrees of deprivation in the economies, resources and population of the surrounding cities, such that these cities play a beneficiary role in the spatial correlation network of the UECC in the YRB.

#### 5.1.2. Closeness Centrality

The average closeness centrality of the spatial correlation network of the UECC in the YRB is 56.262, and 11 cities have numbers higher than the average. This indicates that these cities, as the central ones, have better and faster connections with other cities. Among these cities, there are eight cities, including midstream cities such as Baotou and Ordos, and downstream cities such as Dongying, Qingdao, Zibo, Weihai, Jinan and Yantai, with closeness centralities greater than 90%. These eight cities are the closest to other cities in the correlation network, and they occupy the center of the overall correlation network. Additionally, it is worth noting that the spatial distribution of 11 cities of which the closeness centralities are higher than the average value are quite similar in their spatial distribution to the point centrality. Most of them—such as Ordos, Hohhot, Dongying, Qingdao, Zibo, Weihai and Zhengzhou—are in the midstream and downstream of the YRB. The high closeness centrality of these cities may be attributed to the improvement of the infrastructure, superior geographical location, high economic development level, and good environmental awareness. Moreover, the upstream cities of Lanzhou, Qingyang, and Jiayuguan, and the downstream city of Linfen near the center of the ranking are relatively backwards, indicating that the most remote or edge cities are in the network. These cities are limited by water shortages, land desertification, and serious soil erosion; their UECCs are not high, and they are located at the edge of the network.

#### 5.1.3. Betweenness Centrality

The average value of the spatial correlation network of the UECC in the YRB is 1.004; there are nine cities, such as the midstream cities of Baotou and Ordos, and the downstream cities of Dongying, Qingdao, Weihai, Zibo, Yantai, and Jinan, which are higher than the average. These cities are located in the center of the key nodes in the network, and they play the roles of a “bridge” and an “intermediary”. They have strong control over the entire network. Once these key nodes have problems in the network, the “structural hole” phenomenon will occur, and the network will break. The upstream cities of Lanzhou, Xining, and Yinchuan; the midstream cities of Bayannaoer, Linfen, Shuozhou, and Ulanqab; and the downstream city of Binzhou rank behind the intermediary center, are vulnerable to the intermediary center of the larger cities, and have difficulties controlling and dominating the UECCs of other cities. The betweenness centrality of cities in the network shows obvious unbalanced characteristics. Most of the central cities of urban agglomerations are located in a significant central position in the network and have a good controlling effect. The remaining cities are basically in marginal positions, and are vulnerable to the influence of cities with large betweenness centralities.

### 5.2. Block Model Analysis of the UECC in the YRB

**Figure 7:**
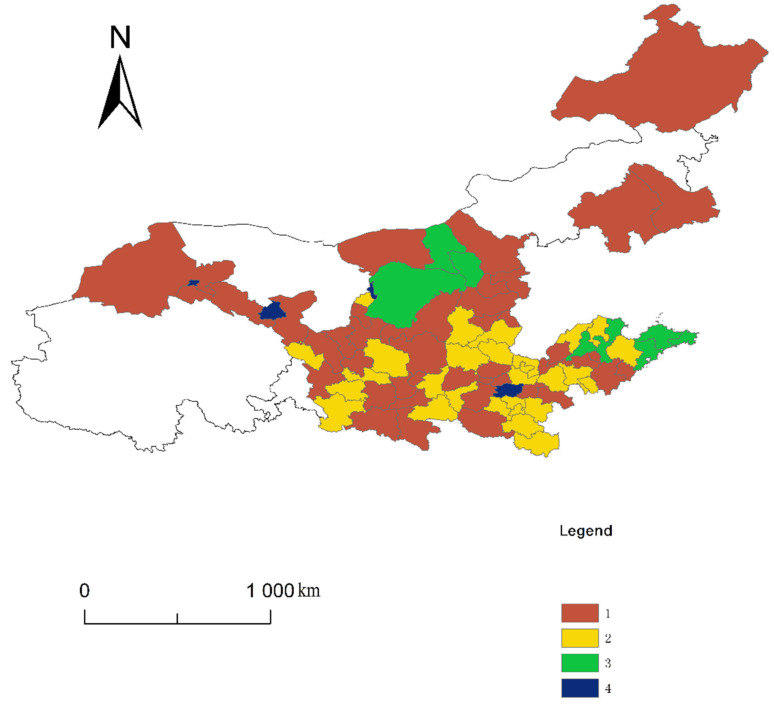
The plate distribution map of the UECC in the YRB.

In order to further understand the structural features and interaction relations of the association network of the YRB, taking 2017 as an example, an in-depth analysis was carried out through the block model. Here, 82 cities in the YRB were divided into four plates, and the CONCOR method in UCINET 6.0 (Analytic Technologies, Lexington, KY, USA) was used for the specific operation. This method is also called the iterative correlation convergence method. The block model analysis can be performed through multiple iterations to find the equivalent points of the location structure in the network. The largest cutting depth was set as 2, and the centralized standard was set as 0.2. Then, ArcGIS10.3 (Environmental Systems Research Institute, Inc., Berkeley, California, USA) was applied to the map, and the spillover effects of the four plates were calculated. According to Figure 7, the first plate mainly includes Ankang, Bayannaoer, Baiyin, Baoji, and 41 other cities; the second plate includes 28 cities, such as Anyang, Binzhou, Dezhou, and Heze; the third plate mainly includes nine cities, such as Baotou, Dongying, Ordos, Hohhot, Jinan, and Qingdao; and the fourth plate includes the four cities of Jiayuguan, Jinchang, Wuhai, and Zhengzhou.

According to Table 4, the total number of relationship networks of the UECC in the YRB in 2017 was 1863, and the spatial correlation and spillover effect between the plates were obvious. The number of relationships between the plates was 1489, and the number of internal relationships between the plates was 374. Specifically, the total number of relations for the first plate was 432, the number of outgoing relationships from within the plate was 110 and the number of external relations sent to other plates was 322. The actual proportion of internal relations was 25.463%, and the expected proportion of internal relations was 49.383%. From this perspective, the number of relations from the first plate to the remaining plates was far greater than that received by other plates. Its net spillover effect was considerable, so it was a typical “net spillover plate”. The total number of relations for the second plate was 285. The number of internal relations from the plate was 55, and the number of relationships received from other plates reached 230. The actual proportion of internal relations was 19.298%, and the expected proportion of internal relations was 33.333%. Therefore, the plate has relations to and receives relations from other plates; however, the number of internal relationships was not large, making this plate a typical “broker plate”. This plate plays a role in connecting the relations of other plates as an intermediary and bridge in the network. The total number of relations for the third plate was 140. The number of relations from within the plate was 17, and the number of external relations received from other plates was 123. The actual proportion of internal relations was 12.143%, and the expected proportion of internal relations was 9.877%. The number of relations received by this plate from other plates was significantly higher than the number of relations sent outward. The spillover effect of the members within the plate was not strong, and the number of external relations received from other plates was significantly more than the number of relations sent out by the plate; therefore, the plate was a “net inflow plate”. There were 72 outgoing relationships for the fourth plate, of which there were five outgoing relationships within the plate and 67 outgoing relationships received by the plate. The actual proportion of internal relationships was 6.944%, and the expected proportion of internal relationships was 3.704%. The number of relationships the plate received from other plates was far greater than the number of relationships of the plate. Therefore, the fourth plate was the “broker” plate.

**Figure 8:**
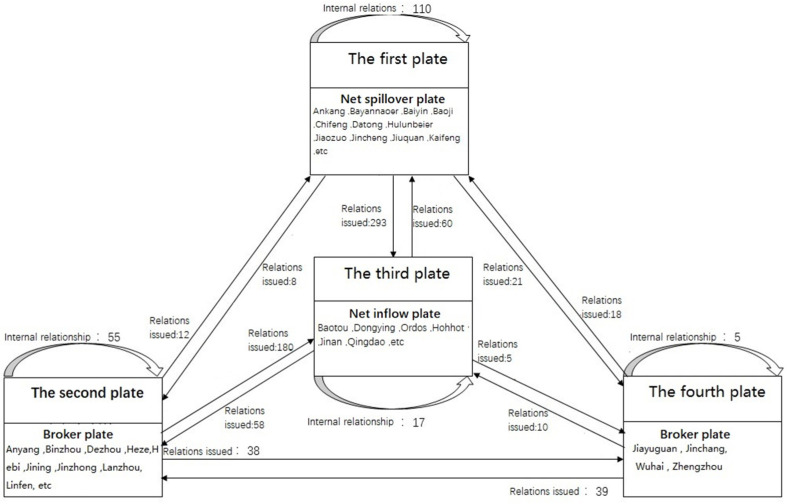
Correlation diagram of the four plates.

The relationship and spillover path between the urban plates of the UECC was further analysed based on the network density matrix calculation. The network density of the spatial correlation of the UECC in the YRB in 2017 was 0.1399. If the value of the plate’s density is higher than 0.1399, it is higher than the overall average, indicating that the UECC of the plate’s cities shows a centralized trend. The density matrix was transformed into an image matrix to intuitively illustrate the correlation and transmission mechanism between the UECC plates (Table 5). The specific operation is as follows: if the value of plate density network is higher than that of the entire network, the value is 1; otherwise, the value is 0. Therefore, a correlation diagram reflecting the correlation between the four plates more intuitively was drawn (Figure 8).

Overall, each plate can play to its advantages and reflect its function. Specifically, the first plate is mostly the central cities of the YRB or the surrounding cities of provincial capital cities. This plate can provide energy, sites and other conditions for ecological protection and sustainable development, and can undertake the transfer of some high-pollution industries. The first plate plays the role of the first engine, and provides the impetus for other plates. Therefore, the first plate is the “net spillover” plate in the network. The third plate has an internal pointing relationship, and receives the spillover relationships of the other plates. This phenomenon occurs because part of the cities contained in the third plate are the central cities or provincial capital cities in the lower reaches of the YRB with a large population and a developed economy, which deprives the surrounding cities of different degrees of energy consumption, ecological environment and economic development. Therefore, the third plate plays a beneficiary role in the network and is the “net inflow” plate. The second plate and the fourth plate play the role of bridges. The main reason is that these plate cities played to their respective advantages in improving the overall UECC of the YRB, greatly promoting the linkage development of ecological environment protection and economic development among the cities in the YRB, and promoting the improvement of the UECC of the entire basin. Therefore, they can become the link and support of the network as the “broker” plate.

## 6. Research Conclusions and Policy Recommendations

### 6.1. Research Conclusions

Based on the panel data of 82 cities in the YRB from 2008 to 2017, the entropy method was adopted for the calculation of the UECC index. The spatial characteristics of the UECC index of the cities was explored using spatial, global and local autocorrelation models. Furthermore, the spatial correlation network of the UECC index was constructed using the modified gravity model, and the structure features of the spatial correlation network of the UECC of the cities were analysed using the social network analysis method. The findings were as follows.

First, in general, in terms of the temporal and spatial evolution of the UECC index in the YRB, from 2008 to 2017, the UECC index of 82 cities in the YRB showed a steady upward trend, showing strong spatial non-stationarity. This shows that the various ecological restoration and protection measures, and the various measures to enrich the people implemented by China in the YRB have achieved remarkable results in recent years, and the UECC index in the YRB is steadily improving. Regarding the spatiotemporal characteristics, the global Moran’s I index of the UECC in the YRB from 2008 to 2017 indicates that the UECC index of the YRB was negatively spatially autocorrelated, and that there was a geographical dispersion effect. It shows that, on the whole, the spatial relevance of the UECC index of the YRB is poor. The value of a city’s ECC index will be negatively affected by the surrounding cities. The hot spots are concentrated in the midstream and downstream of the YRB, including some central cities and provincial capital cities; the cold spots are distributed in the upstream. This shows that the spatial agglomeration of the UECC index in the middle and lower reaches of the YRB is more significant. The cold/hotspot pattern changed greatly.

Second, the total number of spatial network correlations of the UECC in the YRB rises according to the overall network structure characteristics. The degree of network correlation is between 0.8794 and 0.9637. The spatial correlation and linkage effect are not obvious, and the network density is low, indicating that there is a great deal of room for improvement regarding individual network structure characteristics. Cities with higher point centrality and closeness centrality have a consistent spatial distribution, and are mostly distributed in the midstream and downstream of the YRB. The betweenness centrality of each city in the UECC of the YRB shows obvious nonequilibrium characteristics. The central cities of most city agglomerations in the midstream and downstream of the YRB area have a significant central role in the network, and have a strong control role in the network. The remaining cities are basically at edge positions in the network, and are vulnerable to the influence of cities with a larger betweenness centrality.

Third, the analysis of the block model shows that the four plates of the spatial network of the UECC in the YRB are quite different. The number of relationships between the plates is significantly greater than the number of relationships within the plates, and each plate has advantages and functions. The first plate is principally distributed in the midstream and upstream of the YRB, and plays the role of the first engine by providing the power to develop other plates. This plate is the net spillover plate. The third plate has a sporadic distribution in the upstream and downstream of the YRB, and accepts the spillover relationships of the first, second and fourth plates; however, the number of relationships is relatively small. This plate is a net inflow plate. The second plate is principally distributed in the midstream and downstream. The fourth plate is dispersed and scattered in the upstream, midstream and downstream. The second plate and the fourth plate have spillover relations with the third plate while receiving other spillover relations, which means that these plates play pivotal roles in the entire spatial network, and are typical broker plates.

### 6.2. The Policy Recommendations

Based on the above research, in order to comprehensively improve the level of the UECC in the YRB and lay a good ecological foundation in cities in the YRB, the following suggestions are proposed.

First, according to the research conclusions, spatially, the UECC and ecological plasticity in the YRB had strong spatial heterogeneity during the research period, while the spatial heterogeneities of ecological support and ecological pressure were not obvious. The level of the UECC and ecological plasticity of cities in the downstream of the YRB are relatively better than those in the midstream and upstream. Over time, the UECC of most cities has steadily increased, and their subsystems have also changed to varying degrees. Some cities upgrade faster and across multiple intervals. Therefore, in order to promote the overall improvement of the UECC in the YRB, attention must be paid to the various natural environments of the cities in the upstream, midstream and downstream of the YRB, and ecological environmental conservation and governance must be effectively promoted. Therefore, the diverse natural and economic factors in the upper, middle and lower reaches should be fully considered in the ecological conservation and high-quality development of the YRB. Adhering to local conditions, classifying policy, improving the economic and population carrying capacity, and intensifying the high-quality development in the entire watershed are important strategies. For upstream cities, the main objectives are to follow the laws of nature, restore the ecosystem, and reduce human activities’ destruction. First, it is necessary to promote major ecological protection and restoration projects, such as the strengthening of the protection of the main water supply areas in the upstream of the Yellow River, returning farmland to forest, and returning grazing land to grassland. Second, we must address the relationship between the ecological environment and people’s lives and production, abide by the ecological red line in various regions, and reduce the damage caused by overgrazing, excessive tourism and the overexploitation of resources. For midstream cities, we need to strengthen the protection of the soil and water loss, and cultivate characteristic agricultural industries with efficient water saving. For downstream cities, it is essential to enhance the administration of wetland ecosystems in the Yellow River Delta, coordinate flood control safety and high-quality land protection, enhance the economic and population carrying capacity of urban agglomerations, and promote the efficient flow of land, funds, talent and other production factors in the entire basin so that the ecology and environment of various cities in the YRB enter a virtuous circle.

Second, according to the research conclusions, the overall network density of the spatial correlation network of the UECC in the YRB is relatively low, and there is a great deal of room for improvement. The intermediary centrality of the UECC at various levels in the YRB shows obvious non-equilibrium characteristics. In order to solve this problem, it is necessary to improve the connectivity level between the cities in the upstream, midstream and downstream of the YRB, strengthening the entire basin’s coordination and cross-domain linkages, and accelerating network layout construction. First, the government should consider the impact of the urban spatial correlation on the improvement of the environment of the entire YRB, and should commit to improving the density and robustness of the overall spatial network. Second, in the spatial correlation network, with the purpose of realizing the harmonious development of the locality’s UECC, it is necessary to strengthen the ecological linkage between cities in the spatial correlation network from the global perspective. First, we need to focus on promoting the infrastructure construction of cities; realizing interconnections; improving transportation, energy, internet and other important infrastructure constructions; and promoting the convenient flow of population materials and information in the region. By strengthening the construction of the high-speed railway and the Yellow River channel, the level of interconnection of resources and energy is improved. Second, we should build high-quality urban agglomerations along the Yellow River, enhance the carrying capacity of the economy and population of large cities, prevent the unlimited expansion of cities, create a high-quality growth pole of economic development, fully display the demonstration and guidance of big and medium-sized cities in the ecological environment protection and economic development model, and promote the overall improvement of the UECC in the YRB.

Third, we need to formulate differentiated ecological protection policies according to the different plates, and promote cities to open and cooperate in the YRB. Given the different statuses of each city in the interconnected network and the different functions of each plate, more accurate and differentiated ecological protection construction policies are introduced according to local conditions. First, For the net inflow plate, it is necessary to solve its core ecological problems, such as the orderly and effective development of the Shanxi and Ordos Basins as the main energy-rich areas, the Hetao Plain and the Huang-Huai-Hai Plain. The plain is the main carrier of the grain production areas. Second, we must give full play to the spillover effect to help the surrounding cities solve problems related to environmental pollution and insufficient resources, and lay the foundation for other cities to improve their UECC by consolidating food and energy security. For the net spillover plate and broker plate, while making full use of the “engine” and “intermediary” roles, the two-way spillover effect between them and other plates should be strengthened, and the co -protection of the ecological environment and environmental pollution should be strengthened. Therefore, the state should strengthen the construction of high-quality development cooperation zones in Zhengzhou, Luoyang and Xian; jointly strengthen the ecological protection cities and the carrying capacity of resources and the population; promote the overall improvement of the ecological environment system; It also turns the YRB into a significant ecological barrier and a more stable economic zone in China.

## Figures and Tables

**Table 1:** Literature review of the UECC in the YRB.

Research Perspective	Details	Source
Natural ecological environment of the YRB	water resource management	[1]
	sediment sources	[2]
	climate change	[3]
Social ecological environment of the YRB	urban agglomerations along the YRB	[4]
	sustainable development	[5]
	coordinated economic and ecological development	[6,7]
	industrial green development	[8]
Ecological quality assessment of the YRB	Comprehensive ecological environment quality	[9]
	Comprehensive Evaluation of Wetland Ecological Quality	[10]
	the environmental quality of the middle and upper reaches	[11]
	Evaluation of Urban Resilience in the YRB	[12]
	the quality of rivers.	[13]
	soil environmental quality	[14]
Space-time analysis and spatial evolution of the YRB	spatial patterns of the land use	[15]
	spatial matching of water resources	[16]
	spatial matching of sedimentation	[17]
	spatial pattern analysis of the energy eco-efficiency	[18]
	spatial pattern analysis of driving mechanism of urbanization development	[19]
	spatial network characteristics of urban resilience	[20,21]

**Table 2:** Comprehensive evaluation index system of the UECC in the YRB.

Target Layer	Subsystem Layer	Criterion Layer	Index Layer	Index Properties	Mean
UECC	Ecological resilience	Resource supply	Per capita water capacity	+	0.0185
			Garden green area	+	0.0368
			Land productivity = GDP/cultivated land area	+	0.0049
			Per capita land area = cultivated land area/total residential population at the end of the year	+	0.0300
	Ecological support capacity	Environmental protection	The comprehensive utilization rate of industrial solid waste	+	0.0199
			Biosafe disposal ratio of domestic garbage	+	0.0219
			Green coverage in constructed region	+	0.0232
			Urban sewage treatment rate	+	0.0249
		Social progress	Doctors per 10,000 persons	+	0.0197
			Number of college students per 10,000	+	0.0033
			the acreage of streets at year end	+	0.0019
			Number of internet users per 10,000 persons	+	0.0026
			The number of books per 10,000 public libraries	+	0.0068
			Total postal business	+	0.0272
		Economic development	Urban construction land proportion	+	0.0155
			Total retail sales of social consumer goods	+	0.0101
			the increase of GDP	+	0.0557
			The year-end savings balance of urban and rural residents	+	0.0281
			Value Added of Tertiary Industry in GDP	+	0.0224
		Scientific and technological innovation	Science and technology expenditures with respect to local general public budget expenditures	+	0.0057
			Number of practitioners in scientific research, technical services and geological prospecting	+	0.0018
	Ecological pressure	Environmental damage	Industrial sulfur dioxide emissions	-	0.0154
			Industrial wastewater discharge	-	0.0236
			Industrial smoke and dust emissions	-	0.0225
		Resource consumption	Electricity consumption in urban life	-	0.0213
			Total supply of liquefied petroleum gas	-	0.0225
			Residential water consumption	-	0.0213
			Total annual water supply	-	0.0241
		Population pressure	Population density	-	0.0250
			Natural population growth rate	-	0.0258
			Number of employees in the secondary industry	-	0.0232
			The counts of registered unemployment at Year-end	-	0.0172
		Economic growth	The proportion of added value of the first industry with respect to GDP	-	0.0159
			Volume of highway passenger transport	-	0.0197
			Real estate development investment completion	-	0.0235
			highway freight volume	-	0.0219
			Number of industrial enterprises above scale	-	0.0178

Note: (1) Every data point removes abnormal values by filtration. (2) The nature of the evaluation index is divided into positive (+) and negative (-). The larger a positive index value is, the better the UECC. The opposite is true for a negative index value.

**Table 3:** The overall network structure features of the spatial correlation network of the UECC in the YRB.

Time	Average Path Length	Number of Relationships	Average Number of Relationships	Network Density	Network Correlation
2008	2.258	939	11.4512	0.1414	0.8794
2009	2.258	982	11.9756	0.1478	0.9390
2010	2.243	1025	12.5	0.1543	0.9390
2011	2.173	1033	12.5976	0.1555	0.9512
2012	2.126	1014	12.3659	0.1527	0.9514
2013	2.115	1035	12.6220	0.1558	0.9273
2014	2.118	1085	13.2317	0.1634	0.9636
2015	2.144	1102	13.4290	0.1659	0.9637
2016	2.169	1071	13.0610	0.1612	0.9636
2017	2.162	929	11.3293	0.1399	0.8794

**Table 4:** Linkage effect analysis of the spatial correlation plates of the UECC in the YRB in 2017.

UECC Plate	Total Number of Acceptance Relations (Pieces)		Total Number of Relations Issued (Pieces)		The Expected Proportion of Internal Relations (%)	The Proportion of Actual Internal Relations (%)	Plate Characteristics
	In the Plate	Outside the Plate	In the Plate	Outside the Plate			
The first plate	110	90	110	322	49.383	25.463	Net inflow plate
The second plate	55	105	55	230	33.333	19.298	Broker plate
The third plate	17	483	17	123	9.877	12.143	Net spillover plate
The fourth plate	5	69	5	67	3.704	6.944	Broker plate

**Table 5:** Density matrix and image matrix table.

Plate	Density Matrix				Image Matrix			
	The First Plate	The Second Plate	The Third Plate	The Fourth Plate	The First Plate	The Second Plate	The Third Plate	The Fourth Plate
The first plate	0.067	0.007	0.794	0.128	0	0	1	0
The second plate	0.010	0.073	0.714	0.339	0	0	1	1
The third plate	0.163	0.230	0.236	0.139	1	1	1	0
The fourth plate	0.110	0.348	0.278	0.417	0	1	1	1

## Data Availability

The data presented in this study are openly available in National Bureau of Statistics of China.
